# “Beth”: the development of a digital personalised health record and patient portal for use in clinical practice

**DOI:** 10.1192/bjo.2021.509

**Published:** 2021-06-18

**Authors:** George Gillett, Barbara Arroyo, The Beth Team

**Affiliations:** South London and Maudsley NHS Foundation Trust

## Abstract

**Aims:**

To design & develop a clinically scalable personalised health record and patient portal to;

Improve patient safety through improved communication and information sharing between staff, patients and carers, and improved access to safety plans for patients.

Increase the uptake of virtual appointments and video calls rather than over-reliance on telephone calls for clinical care

Empower patients to access supported self-management and self-directed care using digital resources

**Background:**

Current mental health services often rely on telephone calls, letters, text messages and email, which often repeat information to the detriment of the patient. Likewise, care plans and appointments are given in paper cards, which can be lost or become out-dated. Furthermore, service-users often have no access to curated resources, symptom-tracking tools or ability to document their personal treatment targets in medical notes.

**Method:**

Based on service-user feedback, clinical need and the above aims, a digital personalised health record and online portal was developed for patients to record personal goals & coping strategies, access crisis plans, view appointments, track symptoms, complete clinical assessments, communicate with their care-team and access self-management materials. The tool, ‘Beth’, was named after the Bethlem Royal Hospital and was launched in July 2020 to all patients in the South London and Maudsley Trust.

**Result:**

Across the Trust, the tool currently has 710 active users. Features used include; accessing care plans and safety plans, communicating with care teams, organising and viewing appointments, undertaking clinical assessments to inform measurement-based care, tracking symptoms and progress, developing a secure diary, and accessing free & trusted self-management resources.

**Conclusion:**

We have developed “Beth,” a digital personalised health record and patient portal for use in widespread clinical practice. The tool allows patients to take an active role in their care-planning, enhances communication between patients, carers and clinical teams and may improve service efficiency and patient safety. Future development may customise the tool further to incorporate new features and optimise usability for patients and clinicians alike.

